# Postural, pilo-erective and evaporative thermal windows of the short-beaked echidna (*Tachyglossus aculeatus*)

**DOI:** 10.1098/rsbl.2022.0495

**Published:** 2023-01-18

**Authors:** Christine Elizabeth Cooper, Philip Carew Withers

**Affiliations:** ^1^ School of Molecular and Life Sciences, Curtin University, Perth, Western Australia, Australia; ^2^ School of Biological Sciences, University of Western Australia, Perth, Western Australia, Australia

**Keywords:** monotreme, evaporation, convection, conduction, insulation, thermal imaging

## Abstract

We identify for wild, free-living short-beaked echidnas (*Tachyglossus aculeatus*) a novel evaporative window, along with thermal windows, and demonstrate the insulating properties of the spines, using infrared thermography. The moist tip of their beak, with an underlying blood sinus, functions as a wet bulb globe thermometer, maximizing evaporative heat loss via an evaporative window. The ventral surface and insides of the legs are poorly insulated sites that act as postural thermal windows, while the spines provide flexible insulation (depending on piloerection). These avenues of heat exchange likely contribute to the higher-than-expected thermal tolerance of this species. Our study highlights how technological advances that allow for non-contact measurement of thermal variables allow us to better understand the physiological capacity of animals in their natural environment.

## 1. Introduction

Monotreme and therian mammal lineages diverged between 160 and 250 million years ago [1] and monotremes retain many plesiomorphic anatomical, reproductive and physiological traits not shared with therians. Consequently, their biology provides important insights into mammalian evolution, with the short-beaked echidna (*Tachyglossus aculeatus*) considered a particularly useful living model for proto- or baso-endothermy [2,3]. One ongoing question is whether their perceived poor thermoregulatory ability is primitive or derived. While the echidna's low and variable core body temperature (*T*_b_) can be considered a primitive trait reflecting their early divergence from therians, other thermoregulatory characteristics such as metabolic rate, thermal conductance and evaporative heat loss (EHL) capacity are typically therian [4] after accounting for their lower *T*_b_. Despite this, laboratory studies suggest that echidnas have a low thermal tolerance, with a *T*_b_ of 38°C and air temperature (*T*_a_) of only 35°C considered lethal [5,6].

Early studies suggested that the low thermal tolerance of short-beaked echidnas was a consequence of an inability to enhance heat loss due to a lack of sweating, panting, licking or vasomotor adjustment [5–7] and a reliance on behavioural avoidance of high *T*_a_ [5,8]. Indeed their activity patterns suggest they avoid activity at high *T*_a_ and switch to primarily nocturnal behaviour during summer [9,10]. However, short-beaked echidnas have been observed sheltering in hollow logs where *T*_a_ exceeds the supposed lethal *T*_a_ [11] and there is unequivocal evidence that they can increase evaporative water loss and both wet and dry thermal conductance at *T*_a_ ≥ 32.5°C [4]. Although the mechanisms for this enhanced heat loss are currently unknown, thermal and evaporative windows may be important.

Thermal windows are regions of an animal's body surface that vary heat exchange with the environment, being ‘opened’ or ‘closed’ by changes in exposure and/or blood flow; they have been reported for many mammals and birds [12]. Evaporative windows, where endogenous water is behaviourally applied to areas with specialized vasculature, have been reported less often. Forearm licking by kangaroos is one example; saliva is spread over the sparsely furred forearms to evaporatively cool blood in an underlying network of superficial blood vessels [13,14]. Barker *et al*. [4] observed echidnas blowing bubbles from their nares at high *T*_a_ and hypothesized that this enhances EHL. The bubbles broke just behind the nares where the dorsal blood sinus has its maximum blood volume [15]. Here we investigate surface temperatures (*T*_s_) of wild, free-living short-beaked echidnas under a range of environmental conditions to test the hypothesis that the beak tip may act as an evaporative window. We also examine other body regions to evaluate their potential to function as thermal windows.

## 2. Methods

We used infrared thermography [12,16,17] to remotely measure the *T*_s_ of short-beaked echidnas. Echidnas were located at Dryandra Woodland and Boyagin Nature Reserve, in the West Australian wheatbelt, approximately 170 km southwest of Perth. Once sighted, we recorded thermal videos using a FLIR T1050sc infrared camera with an f 83.4 mm 12° telephoto lens, from a distance of 1–20 m. The camera was calibrated against a national standard (FLIR, Oregon, USA). We obtained thermal data in each of 12 months (January to December) for a total of 34 days, during 2020–2021, for 124 echidnas. Most echidnas were not individually identifiable so it was not possible to exclude potentially repeated measurements of some individuals on different days, but recordings were made over a large area in both reserves to minimize the repeated measurement of individuals. Microclimate variables (ambient temperature, *T*_a_; relative humidity, RH; wet bulb globe temperature, WBGT) were collected in a similar location to the echidna under the same conditions of solar radiation, within 0–20 m of the location the echidna was filmed, with a Kestrel 5400 portable weather station.

**Figure 1.**
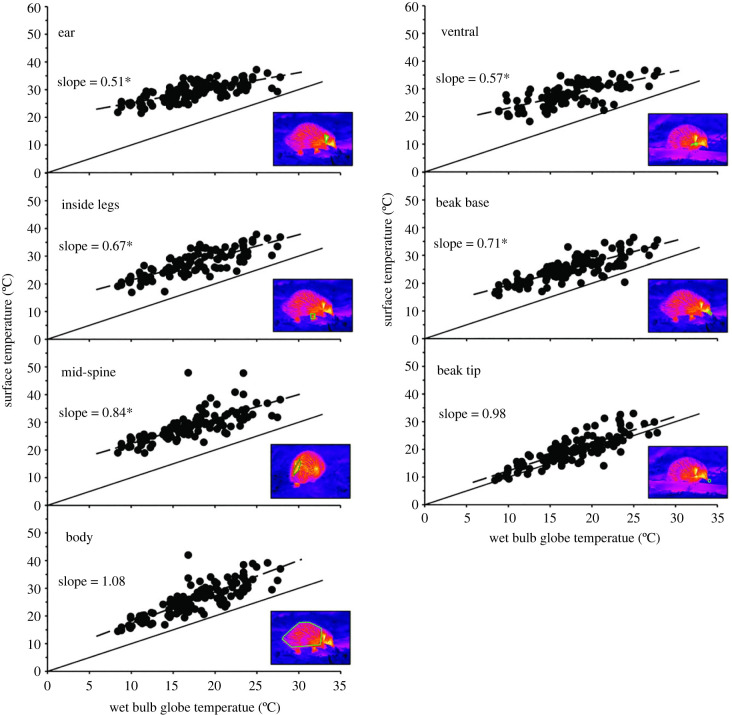
Surface temperature of various body regions plotted against wet bulb globe temperature (WBGT) for 124 active short-beaked echidnas (*Tachyglossus aculeatus*) filmed with an infrared camera in the West Australian wheatbelt. The solid line represents a slope = 1 for WBGT, the dashed line the observed slope for the relationship; asterisks indicate that the slope is significantly different from 1. The inset thermal image shows the body region represented by each panel, outlined with a green polygon.

Thermography videos were analysed using Researcher IR Max4 software, assuming an emissivity of 0.95, with an atmospheric temperature equivalent to *T*_a_, an environmental temperature calculated as the mean of ground and *T*_a_, and the measured RH and camera to echidna distance for each recording (after [17]). Mean *T*_s_ were calculated for polygons manually drawn on video frames showing various body parts; body (lateral and dorsal surface), ventral surface, ear, inside leg, mid-spine (sacral), beak base and beak tip (see insets, figure 1). It was not possible to obtain a *T*_s_ for every body part for every echidna as visibility depended on the animals' orientation, behaviour and the terrain. Ground temperature in the immediate vicinity of each echidna was obtained from the thermal videos.

Statistical analyses were accomplished in RMarkdown [18]. We used a similar approach to Klier *et al*. [19,20] to evaluate the potential for heat exchange of various body regions, by exploring the linear relationship between the various body regions and environmental temperature. Linear mixed-effect models were used to examine the relationship for *T*_s_ with WBGT, body part and observation distance while accounting for individual nested in day as random factors, using the lmer function in lme4 [21] with lmerTest [22] to determine probabilities. Mean *T*_s_ was compared for body parts using the lmer model results using lsmeans with WBGT as an interaction term. As there was a significant interaction between WBGT and body part *T*_s_, we then investigated this relationship individually for each body part. We use WBGT as a measure of the thermal environment for comparison with echidna *T*_s_ as WBGT includes the contributions of *T*_a_ as well as convection, radiation and evaporation to the thermal environment.

## 3. Results

**Figure 2.**
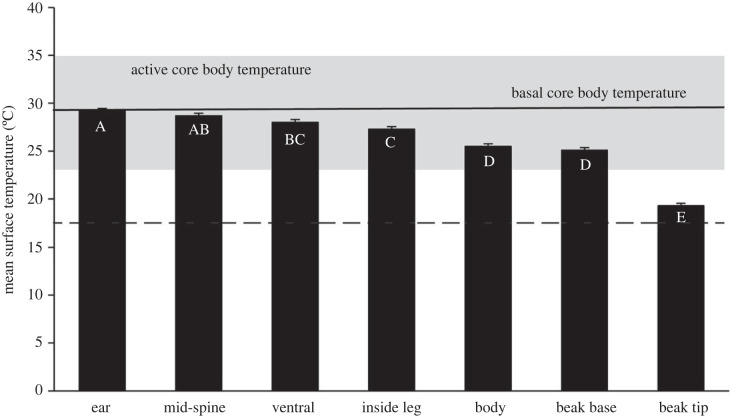
Mean surface temperature of various body regions for 124 active short-beaked echidnas (*Tachyglossus aculeatus*) filmed with an infrared camera in the West Australian wheatbelt. Different letters indicate significant differences between body part surface temperatures. The dashed line indicates the mean wet bulb globe temperature at which echidnas were filmed, the grey area the core body temperature range of active echidnas in a semi-arid habitat [9] and the solid line the basal core body temperature for echidnas from this study site [4].

Videos of echidnas (electronic supplementary material, figure S1) were recorded between 09.56 and 22.41 h, with *T*_a_ ranging from 10.7 to 37.4°C and WBGT from 8.4 to 27.8°C (table 1; electronic supplementary material, table S1). Mean *T*_s_ of the various body parts ranged from 19.3 ± 0.27°C for the beak tip to 29.2 ± 0.27°C for the ear orifice. The mean *T*_s_ of body parts at the mean WBGT (17.3°C) differed (*t*_650–660_ ≥ 4.03, *p* < 0.002), with the exception of ear and mid-spine (*t*_656_ = 1.71, *p* = 0.608), mid-spine and ventral (*t*_658_ = 2.44, *p* = 0.184), ventral and inside leg (*t*_654_ = 2.32, *p* = 0.238), and body and beak base (*t*_656_ = 1.65, *p* = 0.651) regions (figure 2). The *T*_s_ was significantly related to both WBGT (*F*_1,121_ = 38.7, *p* < 0.001) and body region (*F*_6,659_ = 12.8, *p* < 0.001) with a significant interaction (*F*_6,659_ = 5.41, *p* < 0.001), but there was no effect of imaging distance (*F*_1,106_ = 2.88, *p* = 0.093) or of animal nested in day (LRT_2_ = 4.46, *p* = 0.108).

**Table 1.** Microclimate variables collected at the time of each echidna thermal recording with a Kestrel 5400 portable weather station placed in a similar location to the echidna under the same conditions of solar radiation.

	minimum	maximum
ambient temperature (°C)	10.7	37.4
relative humidity (%)	15.8	73.8
wet bulb globe temperature (°C)	8.4	27.8
ground temperature (°C)	7.2	47.0

Analyses for *T*_s_ of individual body parts showed that body *T*_s_ was related to WBGT (*F*_1,52_ = 256, *p* < 0.001) with a slope of 1.08 ± 0.068 and intercept of 6.8 ± 1.23 (figure 1), while the slope for the significant relationship of *T*_s_ of the beak tip with WBGT (*F*_1,38_ = 269, *p* < 0.001) was 0.98 ± 0.060 with an intercept of 2.34 ± 1.08. Neither of these slopes differed from 1 (*t*_38–52_ ≤ 1.20, *p* ≥ 0.096; figure 1). Slopes with WBGT (0.51 ± 0.053 to 0.84 ± 0.068; figure 1) were significant for all other body parts (*F*_1,60–117_ ≥ 51.6, *p* < 0.001), but were significantly lower than 1 (*t*_60–117_ ≥ 2.38, *p* ≤ 0.012; figure 1).

## 4. Discussion

We observed echidnas active at maximum *T*_a_s that were 2.4°C higher than their supposed lethal *T*_a_ of 35°C and up to 5.4°C higher than the previously reported maximum active *T*_a_ [11,23]. Our data, combined with observations that echidnas can shelter in hollow logs at temperatures up to 40°C [11], provide clear evidence that previous estimates of lethal *T*_a_ were underestimates and highlights the value of detailed field studies for evaluating the physiological capabilities of wild animals (e.g. [24–26]). Echidnas can clearly tolerate higher *T*_a_ and are less reliant on behavioural thermoregulation than previously appreciated; we propose that the thermal and evaporative windows we identify here contribute to this thermal tolerance.

The surface temperature of animals obtained by thermal imaging can vary considerably from *T*_b_ [12]. Ear temperature of echidnas most closely approximated *T*_b_ (figure 2); basal *T*_b_ for this sub-species is 29.5C [4] and varies from 23–35°C when active in a semi-arid environment at *T*_a_ from 15–33°C [9]. Consequently, the slope for ear temperature against WBGT was significantly lower than 1, reflecting the considerable effect of *T*_b_ on *T*_s_ for this region. Tympanic membrane temperature, accessed via the external auditory canal, provides the anatomically closest non-invasive access for measurement of hypothalamic temperature [27]. As monotremes lack external ear pinnae, the temperature of the auditory canal can be seen by thermography via the external ear opening.

We identify ventral, inside leg and mid-spine areas as avenues of conductive and convective heat loss that can therefore function as thermal windows. Temperatures of these regions approximated ear temperature (and so are also close to *T*_b_), and the WBGT relationship for ventral and inside leg surfaces also had a slope significantly lower than one, indicating limited insulation. Ventral and inside leg surfaces are sparsely furred, lack spines and can be ‘opened’ or ‘closed’ as thermal windows by postural changes and pressing them against cool soil, a potential heat sink identified by previous workers [11,23]. Their ventral position also protects them from incident solar radiation which is important for maximizing their effectiveness [14].

The echidnas’ dorsal and lateral body *T*_s_ was significantly lower than the body parts identified as thermal windows and scaled with WBGT (slope = 1; albeit with a substantial intercept) suggesting that its surface is well insulated from *T*_b_ and is influenced by environmental conditions such as warming with solar radiation. The body surface is insulated by a combination of a fat layer overlain by a large sub-cutaneous muscle into which the spines are embedded [2]. These three layers clearly provide excellent insulation and explain why Tasmanian echidnas (*T. a. setosus*), with considerable fur between the spines, have similar insulation at low *T*_a_ to the dorsally furless *T. a. acanthion* [28]. However, *T. a. acanthion* can achieve higher thermal conductance at high *T*_a_ than *T. a. setosus* [28] suggesting that the spines provide greater insulative flexibility than fur. The mobility of the spines means that they can be flattened to cover the dorsal surface, ‘closing’ the thermal window and reducing heat loss, or erected, exposing the underlying skin, especially along the sacral (mid-spine) region and ‘opening’ this thermal window. The mid-spine sacral region *T*_s_ was statistically indistinguishable from the ear temperature and significantly higher than the body *T*_s_, but had an intermediate and more variable relationship with WBGT compared to that of the body surface and the ear (figure 1). Incorporating all the dorsal fur into spines provides *T. a. acanthion* with a flexible thermal window to control heat loss in the highly variable thermal environments this sub-species inhabits.

We identify the beak tip of short-beaked echidnas as a unique type of evaporative window. The beak tip, containing a large dorsal blood sinus [15], is kept moist to facilitate electroreception [29]. An additional role of this moist surface is evaporative cooling of the underlying blood within the sinus; with a slope equivalent to 1 and minimal intercept, the beak tip functions as a wet bulb globe thermometer. At high *T*_a_ echidnas blow mucus bubbles, adding moisture to the beak tip [4]. This unique nasal evaporative window is of particular value for echidnas (which do not pant, lick or sweat [5–7]) especially under conditions where environmental temperature exceeds *T*_b_ and evaporation is the only avenue available for heat loss. Although application of exogenous water to the body surface is a widely employed thermoregulatory strategy for mammals, especially those that lack or have few sweat glands [28], there have been few descriptions for endotherms of specialized evaporative windows where endogenous water is behaviourally applied to areas with specialized vasculature. The classic examples of evaporative windows are for storks and turkey vultures, which urinate on their legs that contain extensive subcutaneous vascularization, facilitating EHL [30–32]. Seals on rocks similarly urinate to wet their ventral surface and vascularized flippers to enhance EHL [33], while the licking of vascularized forearms by macropods [13,14] is the best-known mammalian example. Here we demonstrate the utility of the echidnas' novel nasal evaporative window, moistened by nasal mucus bubbles at high *T*_a_ [4], in functioning as a wet bulb that maximally dissipates evaporative heat.

## 5. Conclusion

Echidnas have a more sophisticated suite of thermoregulatory strategies than has been generally appreciated, and we add to this evidence of a novel nasal evaporative window, along with postural thermal windows, and spines that provide for flexible insulation. These physiological strategies explain observations of echidnas active over a much wider range of thermal conditions than has been previously reported and provide mechanisms for how they survive temperatures above their supposed lethal limits. These thermoregulatory traits enable echidnas to inhabit all terrestrial habitats on the Australian continent; they are the most widespread native Australian mammal and have not suffered the same pattern of decline as other critical weight-range (35–5000 g; [34,35]) and arid-zone terrestrial mammals [9].

## Data Availability

The datasets supporting this article have been uploaded as electronic supplementary material [36].
